# Emotional and cognitive changes surrounding online depression identity claims

**DOI:** 10.1371/journal.pone.0278179

**Published:** 2022-12-01

**Authors:** Laura Biester, James Pennebaker, Rada Mihalcea

**Affiliations:** 1 Department of Electrical Engineering and Computer Science, University of Michigan, Ann Arbor, Michigan, United States of America; 2 Department of Psychology, The University of Texas at Austin, Austin, Texas, United States of America; University of Vermont, UNITED STATES

## Abstract

As social media has proliferated, a key aspect to making meaningful connections with people online has been revealing important parts of one’s identity. In this work, we study changes that occur in people’s language use after they share a specific piece of their identity: a depression diagnosis. To do so, we collect data from over five thousand users who have made such a statement, which we refer to as an identity claim. Prior to making a depression identity claim, the Reddit user’s language displays evidence of increasingly higher rates of anxiety, sadness, and cognitive processing language compared to matched controls. After the identity claim, these language markers decrease and more closely match the controls. Similarly, first person singular pronoun usage decreases following the identity claim, which was previously previously found to be indicative of self-focus and associated with depression. By further considering how and to whom people express their identity, we find that the observed longitudinal changes are larger for those who do so in ways that are more correlated with seeking help (sharing in a post instead of a comment; sharing in a mental health support forum). This work suggests that there may be benefits to sharing one’s depression diagnosis, especially in a semi-anonymous forum where others are likely to be empathetic.

## Introduction

The anonymity provided by the internet has made it easier for many people to seek advice on stigmatized parts of their identity. A byproduct of this process is an increasing number of *identity claims*, in which a person shares a specific facet of their identity with a new audience. Social media sites are scattered with identity claims, as people seek to provide information about their identity as context; in lieu of traditional reporting mechanisms like surveys, identity claims found using pattern matching have been used to label users with demographic information such as gender and age [1–3] and mental health status [4–6].

We borrow the term *identity claim* (which we abbreviate as IC) from Gosling, who introduced the term [7]. Gosling’s subsequent work defines identity claims as “symbolic statements made by individuals about how they would like to be regarded” and states that such claims may either be implicit (e.g., displaying a “coexist” bumper sticker) or explicit (e.g., stating “I have liberal political views”). We consider statements made on social media such as “I am a woman” and “I have been diagnosed with depression” to be explicit identity claims. Identity claims on social media can also be implicit; for instance, a woman speaking about her wife indicates that she is part of the LGBT community, and questions about Prozac side-effects indicate that somebody has a mental health diagnosis.

Identity claims are a subset of broader self-disclosures, which have been defined as “The act of revealing personal information to others” [8, pg. 2]; this information need-not be an explicit statement of identity. Disclosure, especially when it relates to stigmatized conditions such as mental health status, can be beneficial, in that it gives people an opportunity to express emotions and develop personal relationships; however, it can also be harmful if the confidant has a negative reaction [9]. The work on self-disclosure is extensive, and multiple theories around self-disclosure exist [10–12].

In this work, we study explicit claims on Reddit of a concealable stigmatized identity, namely a depression diagnosis. We look for patterns such as “I have been diagnosed with depression” which are explicit identity claims and have been used in prior work to identify users with depression on online forums. Examples of identity claims on Reddit that occur in various contexts are given in Table 1. Following work that showed therapeutic effects of schizophrenia self-disclosure on Twitter [13], we first seek to determine if similar longitudinal trends are displayed by Reddit users who claim a depression diagnosis. Given Reddit’s more in-depth posts uninhibited by short character limits and metadata offered by the platform design (e.g., subreddits), we then explore factors of identity claims that correspond with varied changes surrounding those claims. Our research is related both to work in computer science on mental health [4–6, 13–18] and work on identity claims and self disclosure [11, 12, 19–22].

**Table 1:** Paraphrased examples of posts containing identity claims from our dataset.

Example Identity Claim	Context
I need help. A few years ago, **I was diagnosed with depression**, which is common in my family. Anti-depressants helped for a while, but I am no longer able to use them… I have begun to [description of self-harm] again, and I hate doing it but can’t stop. I hate my job, and I have nobody to support me, especially not my family. I simply don’t know what to do. Thank you for reading this…	post in mental health subreddit
Before **I was diagnosed with depression**, I blamed my abilities when I did not perform well at work. However, what you’ve said has made me realize that perhaps my struggles back then were actually caused by my depression, even though I didn’t know it at the time.	comment in mental health subreddit
I have been incredibly bored lately, and following **my diagnosis of depression** a few months ago, staying home all day has triggered my suicidal thoughts. Do you have any suggestions of things to do here in the Des Moines area? It would be especially great to hear about inexpensive activities I could do with my dad, who lives with me.	post in another subreddit
You ex-boyfriend sounds horrible, I’m glad to hear that you were able to get away from him. Based on what you said, it sounds like you have a mental health diagnosis. **I’ve also been diagnosed with anxiety and depression**, and it is great to hear that you are able to get support from group therapy.	comment in another subreddit

The identity claim pattern is demarcated in bold. Examples are given for posts and comments that occur in subreddits with various topics.

In computer science, a significant body of work has emerged that focuses on the objective of identifying mental health status from text [4–6, 23] In this line of work, diagnosed users are usually identified using pattern-matching. A parallel line of work seeks to use text data to better understand various phenomena related to mental health, including but not limited to the effects of participating in discussions around mental health on online platforms. This includes a study of the effects of long-term participation in online mental health communities, which finds improvement in linguistic dimensions including negative emotion and first person singular words [18]. In a study that is most closely related to our own, Ernala et al. [13] study Twitter users before and after reports of a schizophrenia diagnosis, finding significant changes occurring around the report, which we would refer to as an identity claim. Our study differs in that we focus on depression, a condition that is not considered in prior work on the effects of diagnosis disclosure, and we focus on differential longitudinal change and content of identity claims that appear in different contexts, finding that context plays a clear role in the language change surrounding identity claims.

In addition to work applying NLP, AI, and HCI techniques to understand mental health, we are also inspired by a body of work on self-disclosure. Concerns of impression management can inhibit other goals surrounding self-disclosure (emotional support, accountability, motivation, advice), especially on public social media forums that are linked to real-life identities [10]; still, researchers have found evidence of intimate self-disclosures on both anonymous and identified social media.

Significant stress often leads to people opening up on Facebook, which in turn can lead to social support and reduced depression [19], and depression disclosures on Instagram have led to supportive responses [20]. Following the disclosure of people’s transgender identity to people in their offline circles, there was initially an increase in negative sentiment on their Tumblr transition blogs, but this was typically followed in the long-term by an increase in positive sentiment [21]. People tend to prefer disclosing major life transitions (including transgender identity and mental health conditions) to online, anonymous support groups, as the shared experiences in these groups can lead to a stronger emotional bond [22].

Frameworks for self-disclosure have been expanded and created for social media disclosures. A framework of decision factors related to pregnancy loss disclosure was developed based on interviews with women about their self-disclosures of pregnancy loss on social media [11], and includes factors such as self-related, audience-related, and societal. The functional theory of self-disclosure, which helps to describe disclosure goals and functions, can be applied to social media, and those goals vary based on the number of people who will see messages [12]. This work influences how we think about the potential goals of depression identity claims; some goals from the functional theory that may be acted on are identity clarification (defining one’s position for self and others) and self-expression (relieving distress through venting negative emotions and disclosing problems).

### Contributions and hypotheses

We address the following research questions:

**RQ1**: Does a person’s language use change before and after they make a depression diagnosis identity claim, and if so, how?
**RQ2**: How does the way the identity claim is made correlate with the level of change in cognitive and emotional processes?
**RQ3**: How does interaction with mental health subreddits correlate with the changes that are observed?

We hypothesize that the act of clearly identifying oneself with a depression diagnosis is a meaningful event. Disclosure of such an identity opens up the opportunity to receive social support and enables the person making the identity claim to freely express their thoughts and feelings. Disclosure of stigmatized identities (such as mental illness) can also have negative consequences; however, work studying disclosure of such identities has primarily focused on potential negative response of the confidant as a factor leading to negative consequences [9], and we hypothesize that this primarily occurs when the person making the identity claim has an important long-term relationship with the confidant. On anonymous social media platforms where identity claims are commonly made with the objective of seeking or providing support, negative responses seem less likely. We further hypothesize that identity claims that correspond with the largest longitudinal behavioral change will display intentionality in sharing (we formalize this notion by considering the difference between *posts*, in which the user begins a discussion, and *comments*, which appear in a contextual situation initiated by somebody else) and intentionality in support seeking (by posting in subreddits that provide mental health support). In order to measure symptomatic change surrounding identity claims, we can look at how the usage of psychologically meaningful words changes before and after users make an identity claim. Increased sadness, anxiety, cognitive processing, and self-focus are commonly observed in the writing of people suffering from depression; we expect usage of words from these categories to build up in the time before the identity claim (when the users may have already been diagnosed, and are working to understand their identity), and then decrease following their identity claim. Analytical thinking is likely to decrease around the time of the identity claim; this type of language is less personal and more formal [24], and we expect it to become less common as people process their diagnosis. We also examine health-related words, expecting them to spike around the time that people share their diagnosis and decrease in use afterwards; this can be thought of as a sanity check to confirm that there is increased focus on one’s health around the time that they share their medical diagnosis.

Focusing on a cohort of over 5,000 Reddit users, we find significant longitudinal effects as users build up to explicitly sharing their depression diagnosis. Features that typify the language of people who suffer from depression are abundant, especially in the period prior to one’s identity claim; these features tend to drop off following the identity claim, although they do not approach the levels observed in a control group. Furthermore, we find that not all identity claims correspond with equivalent changes in language use, finding more change when the identity claim is made in post rather than a comment and when the identity claim is made in a subreddit that focuses on mental health support. Our results show a strong correlation between identity claims and changes in linguistic attributes that are indicative of depression; however, due to the data we have access to, we cannot make causational claims.

## Materials and methods

### Data collection

We collect data from the social media website Reddit using the Pushshift dataset [25], including data from 2006–2019. Reddit is a social media platform that is separated into multiple “subreddits,” which are topical communities ranging from r/depression (a depression support forum) to r/aww (a forum for sharing cute animal videos) to r/wallstreetbets (a forum for discussing the stock market). Users *post* to subreddits, and other users may *comment* on those posts. Reddit is semi-anonymous, in that each user has a pseudonymous username that need not be connected to their real identity. The anonymity provided by Reddit enables disclosure of stigmatized facets of people’s identities, including but not limited to mental health status [11, 26].

We follow a procedure used to collect datasets for mental health status classification to build a dataset of self-reports of mental health diagnoses [5, 6]. First, we look for diagnosis patterns such as “i am diagnosed with,” excluding posts with negative patterns such as “never been officially diagnos.” The posts are labeled as depression identity claims if the word “depression” or other related words are mentioned within the 40 characters following the diagnosis pattern, using the list of words from [6]. Some examples of phrases matching the patterns are shown in Table 1. Additional examples of phrases that would match the patterns include “my doctor diagnosed me with depression and ADHD” and “I suffer from diagnosed depression.” While some users make implicit identity claims that can be used to infer a mental health diagnosis through common-sense reasoning, we do not attempt to identify those claims, focusing only on explicit statements. Once we collect the list of users, we retrieve all posts and comments from those users.

The pseudonymous nature of Reddit means that data cannot be directly linked to real-world identities; however, it is possible that users *could* include some identifying information in their username. To counteract this possibility, our code for collecting data replaces the original usernames with IDs that cannot be mapped back to the user, following best practices in social media health research [27]. Our study accesses only public data and is consistent with Reddit’s terms of service. We submitted our initial plans to the IRB and were given exempt status (HUM00195968 at the University of Michigan). Since the original data was missing the metadata, we performed our own data collection from Reddit using the same protocol for data collection as the original dataset covered by the IRB. Reddit is a public platform and Reddit usernames are pseudonyms. Still, as mentioned in the text of the paper, we fully anonymized all usernames by replacing them with an ID that cannot be mapped back to the username in our data collection script. We did not seek informed consent as it is atypical to collect consent in retrospective studies using anonymous social media data in which there is no interaction with the users who wrote the text. The initial IRB exemption expected no contact with users.

We use the following fields in our analysis: text (a combination of the anonymized user ID, body and selftext fields for posts), subreddit, and created_utc. For each post/comment, we compute boolean fields that are used to address RQ2 and RQ3: is_comment and is_mh_subreddit. We ignore posts with the text [deleted] or [removed].

Because we aim to capture the average behavior of individual users, we ensure that each user is active across the two-year span that we study (one year before and one year after the identity claim). We filter users for inclusion in our longitudinal analysis as follows:

1. We include only users who are active on Reddit in at least 25% of the one-week periods in the year both before and the year after their identity claim.
2. We include only users whose activity *spans* the entire two-year period. In other words, they must either be active during or before (after) the first (last) week in our target period.
3. We remove users who are *inactive* for more than eight weeks at any point in our target period.

The aim of our filtering procedure is to balance the competing goals of (a) ensuring that the user activity levels allow for robust study of long-term trends and (b) not excluding too many users from the study. In particular, with respect to (a), we want to ensure that we are studying the average long-term behavior of individual users, rather than only looking at users who had data available in each period. If all users were not fairly active before and after their IC, we may end up measuring the difference between users who posted for a long time before making an IC and users who only posted a few times before making an IC, which would not allow us to answer our research questions, pertaining to the behavior of individual people. With this in mind, we implemented rules 1 and 2 above. After finding that there were still significant chunks of missing data when the first two rules were implemented, we also added rule 3, after which whether or not we interpolate missing values, the plots show similar trends.

With respect to (b), when crafting rule 1, we compute the number of users who would be included if we considered various time periods before and after the IC (ranging from 1 month to 3 years). We find that one year and 25% activity achieves our goal of covering a long period of time while still including a large number of users. A very strict activity ratio of 100% one year before and after the IC would lead to an almost 20x reduction of the number of users in the study, whereas keeping the activity ratio at 25% and reducing the activity period to only three months before and after would lead to less than a 1.5x increase in users. Our choice of one year before and after the IC is similar to related work [13], which includes posts 200 days before and 400 days after Schizophrenia disclosures after empirical analyses of posting volume before and after disclosure. The major difference is that we filter users based on activity levels, which is possible given our relatively large dataset.

We remove some users who had excessive amounts of data and were confirmed to be bots following manual examination (e.g., their posts explicitly stated that they were bots or used extremely repetitive language indicative of bots [28]); however, it is possible that some bots may still appear in the dataset. We also exclude four users who cannot be matched to controls using the methodology explained the following section, leaving us with a total of 5,854 users.

#### Control data

We collect data for a control user for each of the users in our dataset. We design our method for matching controls to ensure that there is a general topical and temporal match between our users and the controls. This results in a less biased comparison than if we compare users with radically different posting behavior. While there are no labels on Reddit for properties such as gender and age that would typically be used to match controls in psychological studies, we expect that our topically relevant control group is better matched with respect to demographics than a truly random group of Reddit users.

Our controls are identified using the following steps: First, we identify “time zero” for the control to match the IC timestamp, which removes temporal variability, following [13]. The control users must match all of the filtering requirements for inclusion in longitudinal analysis (active in 25% of one-week periods for the year surrounding time 0, activity spans the two year period, users who are inactive for more than 8 weeks are removed). Second, the controls must have at least one post or comment within one week of “time 0.” Third, all control users that are judged to be valid are scored against the IC based on their subreddit activity. A vector is created to represent each IC user as the percentage of their posts or comments that occur in a set of non-metal-health subreddits. We only consider subreddits that at least 1% of the IC users are active in. The intention of this process is to identify users with similar posting activity to the IC users outside of a mental health context. We then score each IC-control pair using cosine similarity of the vectors. Finally, we choose the control user with the highest score for each IC. This process is greedy; if a control matches to multiple ICs, we choose the IC with the highest similarity, as we only allow 1–1 mappings.

A summary of our data is provided in S1 Appendix. While we do not make the raw dataset available, as it is standard practice in mental health applications due to privacy concerns [5, 6], we share processed data that allows for the replication of the main results in the paper. Specifically, we share LIWC values for all posts and users (IC and control) with time differences from the IC date in addition to counts to replicate the n-gram analysis. For further analysis of the raw text data, the Pushshift dataset from which we obtained our data is readily available [25].

### LIWC

We use the LIWC 2015 lexicon [29] to measure linguistic attributes in Reddit posts. The LIWC lexicon consists of seventy-three hierarchical language categories, encapsulating properties including linguistic categories (e.g., 1st person plural pronouns, verbs), emotions (e.g., anxiety, sadness), time (e.g., present, future), and personal concerns (e.g., work, money, death). In addition to the base categories, LIWC computes summary variables which capture surface-level measures (e.g., words with more than six letters, words per sentence) and variables derived from other categories (e.g., analytical thinking, clout). The LIWC lexicon has been used extensively in work that analyzes mental health using language data, especially from social media [4, 13, 30–35]. In particular, we focus on six categories: cognitive processes, analytical thinking, sadness, anxiety, health related concerns, and 1st person singular pronouns, which have been shown to be substantially different from the general population for those with depression and those experiencing life upheavals in prior work [4, 6, 24, 36–40]. Examples of words that comprise the LIWC categories used in our analysis are given in S1 Appendix.

### Longitudinal analysis

In our work, we aim to see how user’s language changes both in the buildup to and following an identity claim. Our longitudinal analysis captures the average behavior of users who make an identity claim.

We center our time series around the identity claim, which we consider to be taking place at time zero. If a user has multiple identity claims, we center around the *first* of their claims, and we exclude the posts containing identity claims from the rest of our analysis.

Given a metric such as percentage of words in the anxiety category, we first compute $\mu_{u}^{t}$, the average value for the metric for user *u* during week *t*. Next, we average the values across our set of users, such that each point in our time series represents the following value:

$$\begin{array}{c} s^{t}=\frac{1}{\left|users\right|}\cdot \sum_{u\in users}\mu_{u}^{t} \end{array}$$ (1)

To ensure that the same users are represented in each of the data points in our time series (and that the effects we see are not simply a reflection of who is active in each time period), we use linear interpolation on the time series for individual users to fill in missing values; the mean percentage of weeks filled in with interpolation for IC users is 11.47%, while the mean for controls is 16.24%. With the data filtering we performed, we found in early experiments that the results are robust and are similar regardless of whether or not this interpolation is performed. Our data filtering process ensures that the users we study are active over a long time period.

### Interrupted time series analysis

To assess the significance of changes observed in our data, we use interrupted time series analysis [41]. This approach has been used in other studies of changes in language and other properties of online conversations following an event [42–46]. We fit the following regression model:

$$\begin{array}{c} Y_{t}=\beta_{0}+\beta_{1}T+\beta_{2}X_{t}+\beta_{3}TX_{t} \end{array}$$ (2)

Three variables are extracted from observations in our time series:

*Y*_*t*_: the outcome, e.g., % of words in the anxiety LIWC category
*T* ∈ [−52, 52]: the week in the time series
*X*_*t*_: an indicator variable representing whether the observation is before or after the identity claim (in our case *X*_*t*_ = 1 if *T* > 0, and is otherwise 0)

We then learn the coefficients:

*β*_0_: the intercept
*β*_1_: the slope
*β*_2_: the *change* in the intercept following the identity claim
*β*_3_: the *change* in the slope following the identity claim

We are particularly interested in quantifying how the slope changes following the identity claim. Therefore, we report the value of the coefficient *β*_3_ and the p-value associated with that coefficient in our results tables. We fit the model using the Ordinary Least Squares implementation from the statsmodels package [47]. In order to control for multiple comparisons, we use the adjustment for false discovery rate (FDR) [48]. This correction is performed for all results in each table.

### N-Gram analysis

In order to characterize the differences in the content of identity claims that occur in different contexts, we use log-likelihood ratio (LLR) analysis, which has been used in other similar social media studies [31, 49, 50]. First, we compute the frequency of unigrams and bigrams in each of the corpora *A* and *B*, which consist of all identity claims made in the different contexts (for instance, *A* on mental health subreddits and *B* on other subreddits). The n-grams are counted in each post, then summed across corpora. We include only those n-grams that appear in at least ten identity claims in each corpus. Using n-grams that appear in only a small number of identity claims could lead to privacy concerns (n-grams could be searched to identify users), and lead to more noise caused by differing ratios of very infrequent n-grams. We base our threshold on prior work has also used a threshold of 10 for filtering [51]. We then compute the LLR for each n-gram *x* as

$$\begin{array}{c} LLR_{x}=\text{log}(\frac{P(x|A)}{P(x|B)}) \end{array}$$ (3)

A positive LLR indicates that a n-gram is more frequent in corpus *A*, while a negative LLR indicates that the n-gram is more frequent in corpus *B*.

## Results

In order to characterize people’s language use before and after their identity claim, we graph LIWC values [29] corresponding to their Reddit posts and comments spanning from one year prior to one year following their identity claim. These values capture the language dimensions of sadness, anxiety, cognitive processing, analytical thinking, self-focus, and health related concerns. We check for statistical significance of changes and characterize the magnitude of the changes numerically using Interrupted Time Series (ITS) analysis [41]. Finally, we use n-gram analysis to compare the contents of identity claims that occur in different context.

### RQ1: Does a person’s language use change before and after they make a depression diagnosis identity claim, and if so, how?

We first examine how identity claims correspond with abrupt changes in people’s language. We hypothesize that after sharing information about one’s identity, we may see therapeutic effects similar to those observed in [13] that lead to language usage that more closely approximates those without depression, and we compare the language use of those who made identity claims to that of matched controls who we hypothesize should have no change.

**Fig 1:**
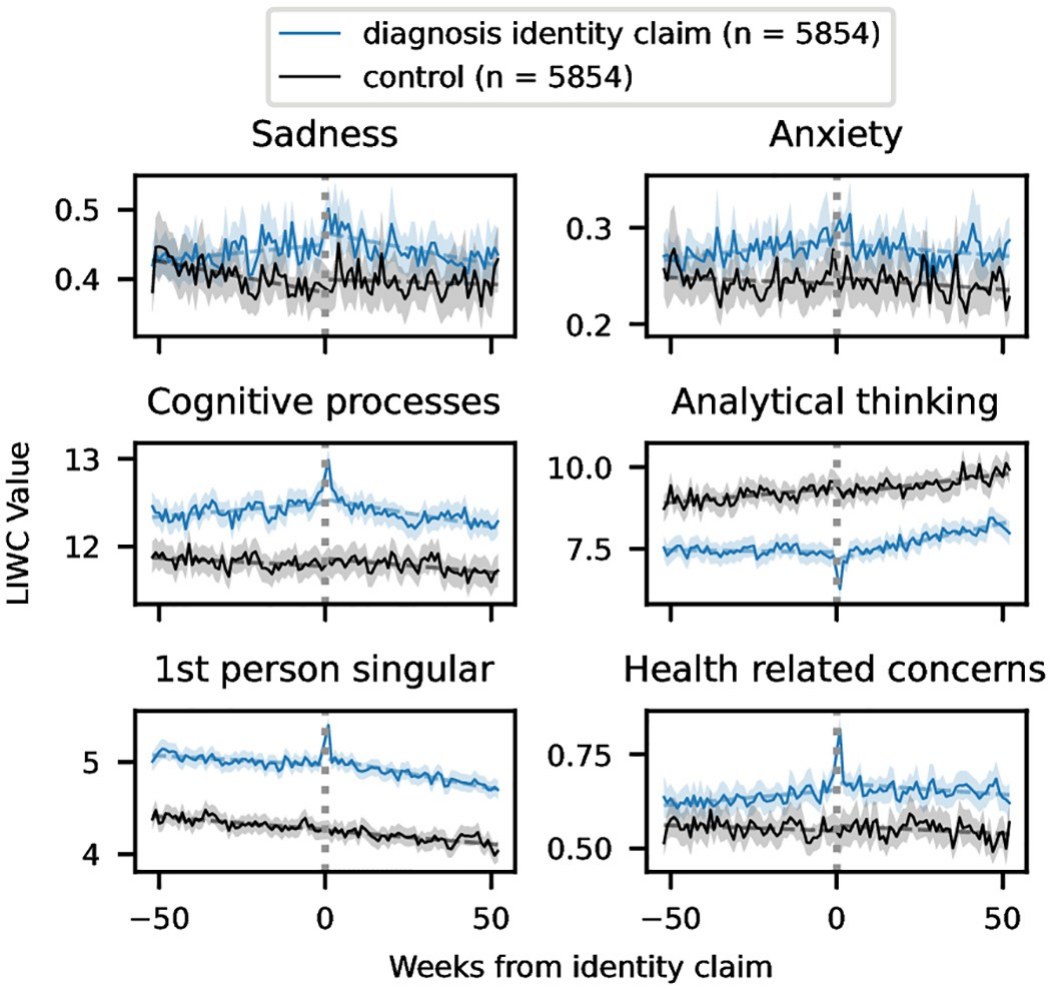
LIWC changes over time for users with a diagnosis identity claim and controls. Analytical thinking is computed based on other categories; the other y axes are percentage of total words. The solid line marks the weekly mean values; the dashed line shows the trend lines for the before and after periods, and the shaded area covers the 95% confidence interval.

The time series plots are presented in Fig 1; we also conduct interrupted timeseries analysis to evaluate the significance in the change in the trend, which is presented in S2 Appendix. We find that there are significant changes (based on p-values ranging from 2.31e-05 to 4.31e-17) in all of the examined LIWC language categories [52] for those who make a diagnosis identity claim; the changes in trend are clearly visible from the graphs. The initial rise in cognitive processing may indicate the process of people working through issues in their lives; this precedes the identity claim and falls off after, which may indicate acceptance of the diagnosis. The opposite pattern is observed for analytical thinking; the increase is positive in that the language use indicates logical and formal thinking, which is necessary for many day-to-day tasks. The steeper drop-off in first-person singular pronouns also has positive implications, as their use indicates self-focus which is a symptom of depression; the self-focus is clearly higher in the depressed users than the controls, but the increased negative slope indicates a lessening of this symptom. Anxiety and sadness are common emotional symptoms of depression; they rise before the identity claim but drop off after, again indicating a lessening of symptoms. Finally, health-related concerns reach their peak around the time of the identity claim, as one would expect. The levels of their discussion remain high throughout the following year, indicating a longer-term commitment to discussing one’s health following the identity claim. The only statistically significant change for the control users occurs in the sadness category; there, we see a steep drop-off initially, followed by leveling off.

We also see that while we exclude the identity claim posts from our analysis, there are clear spikes in most categories around the time of the identity claim. This suggests that discussion of the situation surrounding one’s newly claimed identity extends to other posts in the weeks surrounding the identity claim.

### RQ2: How does the way the identity claim is made correlate with the level of change in cognitive and emotional processes?

We consider how the way in which a user shares their identity corresponds with differences in the temporal trajectory following the identity claim. On Reddit, there are two main ways to interact with the site: posting and commenting. These two methods may correlate with different reasoning for sharing one’s diagnosis: users who share in a post are creating content for others to interact with; some are seeking help. They intentionally share their diagnosis because they think it is necessary to the understanding of people who may read the post. Meanwhile, those who comment are *reacting* to content shared by somebody else. They may mention their identity in reaction to learning about somebody else’s identity; in other words, the identity claim is partially based on context introduced by another person. We hypothesize that the highly intentional identity claims that are produced without initial context will yield more extreme changes over time.

Therefore, we split the users into those whose identity claim is shared in a Reddit post and those whose identity claim is shared in a comment (316 users who have identity claims in both posts and comments were excluded from this analysis). We perform n-gram analysis in order to measure differences in the words people tend to use in identity claims that occur in these two contexts (see Table 2 for full results), which shows stark differences between the two sets of identity claims. Identity claims that occur in posts seek solidarity, help and support from others (“any advice,” “need some [help/advice],” “you guys,” “if anyone”), refer to specific times (“2015,” “2016,” “2017,” “september,” “this week”), and suggest thanks (“[thank you] for reading,” “[thanks] in advance”). Identity claims that occur in comments frequently address the concerns of others (22 of the 25 top n-grams in this category have the word “you” or “your” in them) and express a desire to help (“help you,” “you should,” “think you,” “you might”) along with feelings of solidarity (“like you”).

**Table 2:** Results of our N-Gram (LLR) analysis.

IC Post		IC Comment		IC MH Subreddit		IC Other Subreddit	
n-gram	LLR	n-gram	LLR	n-gram	LLR	n-gram	LLR
for reading	2.11	agree	-1.82	my adhd	2.16	game	-1.28
any advice	1.99	yourself	-1.72	of bipolar	1.89	clinically	-1.08
tonight	1.86	you ll	-1.69	for adhd	1.89	gym	-0.97
in advance	1.84	like you	-1.69	bipolar ii	1.87	fat	-0.93
can seem	1.83	where you	-1.64	does anyone	1.85	chemical	-0.89
2017	1.77	help you	-1.62	have adhd	1.84	team	-0.83
hello	1.74	what you	-1.59	adhd but	1.83	mental illness	-0.81
2015	1.72	your life	-1.56	mania	1.77	baby	-0.81
to post	1.69	are you	-1.54	hyper	1.72	men	-0.80
2016	1.66	you are	-1.50	has anyone	1.69	power	-0.79
advance	1.66	it sounds	-1.50	misdiagnosed	1.67	with clinical	-0.79
september	1.65	to your	-1.49	lithium	1.65	cool	-0.78
need some	1.65	sounds like	-1.46	sertraline	1.65	growing up	-0.77
and hate	1.64	think you	-1.44	diagnosis and	1.64	if was	-0.76
this week	1.63	you should	-1.44	ii	1.63	exercise	-0.75
been having	1.62	you need	-1.42	love me	1.60	op	-0.72
moved in	1.60	in your	-1.40	official diagnosis	1.60	we had	-0.72
wedding	1.58	with you	-1.36	the adhd	1.59	comment	-0.72
is this	1.58	make you	-1.35	few friends	1.55	how you	-0.71
you guys	1.57	you might	-1.35	begun	1.55	talk about	-0.71
hi	1.57	you want	-1.35	atypical	1.53	are you	-0.70
if anyone	1.55	with your	-1.35	worried that	1.53	you ve	-0.70
facebook	1.55	for you	-1.34	this morning	1.51	clinical depression	-0.69
wondering if	1.53	your brain	-1.30	with therapist	1.51	something to	-0.69
as of	1.51	you that	-1.29	psychiatrist who	1.49	clinical	-0.68

We compare identity claims made in posts to identity claims made in comments and identity claims made in mental health subreddits to identity claims made in other subreddits. We show the 25 n-grams with the highest (IC Post, IC MH Subreddit) and lowest (IC Comment, IC Other Subreddit) LLR. High LLR indicates that the n-gram is more likely to appear in an identity claim in a post/mental health subreddit; low LLR indicates that it is more likely to appear in an identity claim in a comment/non-mental health subreddit. Identity claims that occur in posts seek solidarity, help and support from others, refer to specific times, and suggest thanks. Identity claims that occur in comments frequently address the concerns of others and express a desire to help along with feelings of solidarity. Identity claims in mental health subreddits sometimes center around comorbidities, seek advice and solidarity, speak more frequently about the diagnosis, and mention mental health practitioners. The identity claims in other subreddits, however, more frequently specify that the form of their depression is clinical, are part of a conversation with others, and relate their depression to other parts of their life.

**Fig 2:**
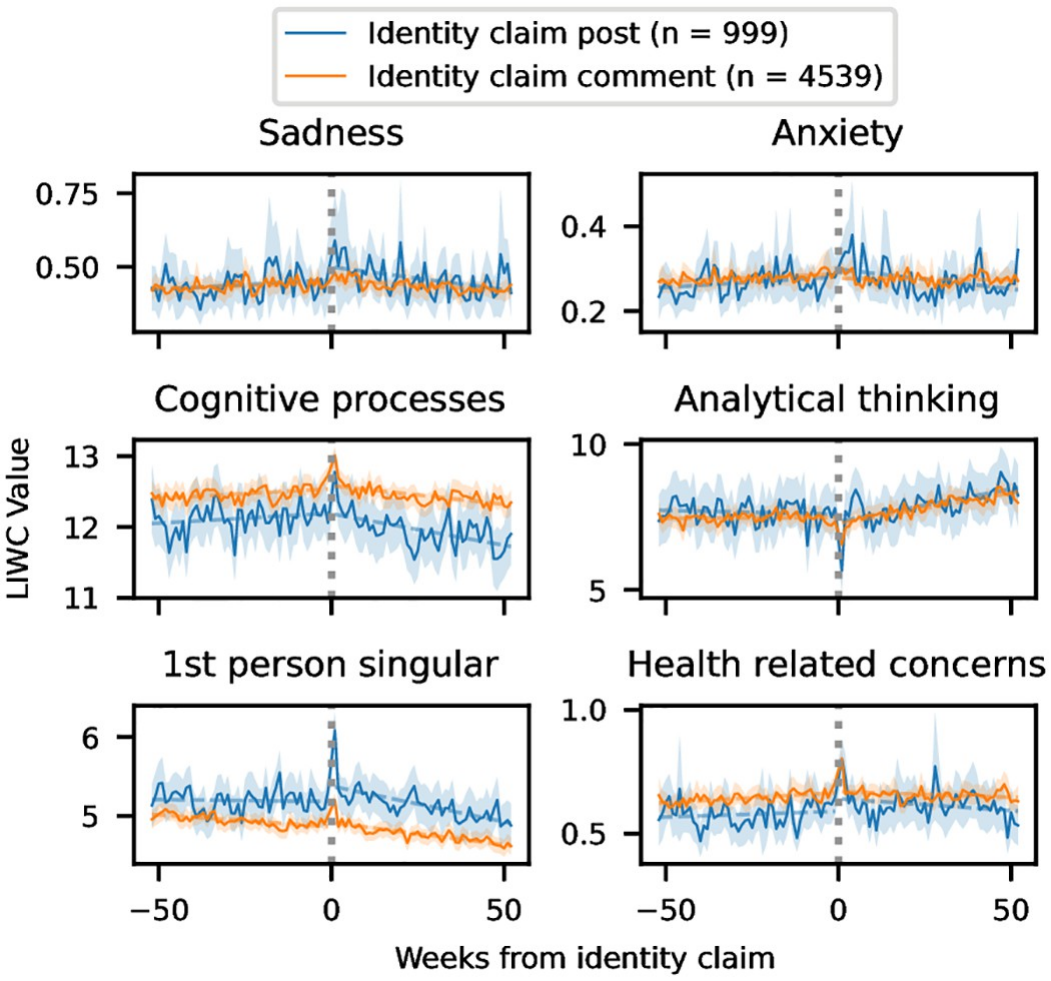
LIWC changes over time for users whose identity claims are in the form of posts and comments. Analytical thinking is computed based on other categories; the other y axes are percentage of total words. The solid line marks the weekly mean values; the dashed line shows the trend lines for the before and after periods, and the shaded area covers the 95% confidence interval.

The time series plots are presented in Fig 2, and the interrupted time series analysis is presented in S2 Appendix. We find that there are significant changes (based on p-values) in all of the examined LIWC categories, **both** for users whose identity claim appears in a post and users whose identity claim appears in a comment. Furthermore, the direction of change is the same for both groups. However, in every linguistic category, we see that the magnitude of change is larger for users whose identity claim appears in a post. In the graphs, we also observe larger spikes in the week that directly follows the identity claim for users who share their identity in a post. For most of the linguistic categories, we see a clearly positive slope in the buildup to the identity claim, followed by a decrease, however this is not universally true. The slope of analytical thinking for users who make identity claims in posts is slightly negative, while it is slightly positive for users who make an identity claim in a comment. We then see a significant change to a steeper positive slope for both groups following the identity claim. Meanwhile, while the upward slope nearly vanishes, discussion of health does not decrease during the year following the identity claim for users whose identity claim in in a comment. Finally, for users whose identity claim is in a post, the slope of first-person singular pronouns is almost zero for the first year. This is an interesting result, as it differs from the full set of users with an identity claim.

### RQ3: How does interaction with mental health subreddits correlate with the changes that are observed?

We similarly believe that *where* a user shares their identity is indicative of intentionality. When a user’s identity is shared on a subreddit that focuses on mental health, they may be seeking support from a network of peers who have shared experiences [22]. Seeking out this network indicates that they may consider their depression diagnosis to be a more core aspect of their identity, compared to users who may share their experiences with, for instance, a subreddit comprised of students from their university. Even if a user shares their identity claim in a comment, we hypothesize that doing so in a mental health subreddit signals intentionality.

We begin by performing n-gram analysis to examine the linguistic differences between identity claims that occur in mental health subreddits and other subreddits (see Table 2 for full results). We exclude 311 users from this analysis who have identity claims in both mental health and other subreddits. Identity claims in mental health subreddits sometimes center around comorbidities (n-grams containing “adhd,” “maina,” and “bipolar”), seek advice and solidarity (“does anyone”, “has anyone”), speak more frequently about the diagnosis (“misdiagnosed,” “official diagnosis,” “diagnosis and”), and mention mental health practitioners (“with therapist,” “psychiatrist who”). The identity claims in other subreddits, however, more frequently specify that the form of their depression is clinical (“clinically,” “with clinical,” “clinical depression”), are part of a conversation with others (“how you,” “are you,” “you ve”), and relate their depression to other parts of their life (“gym”, “fat”, “baby”, “exercise”, “growing up”).

**Fig 3:**
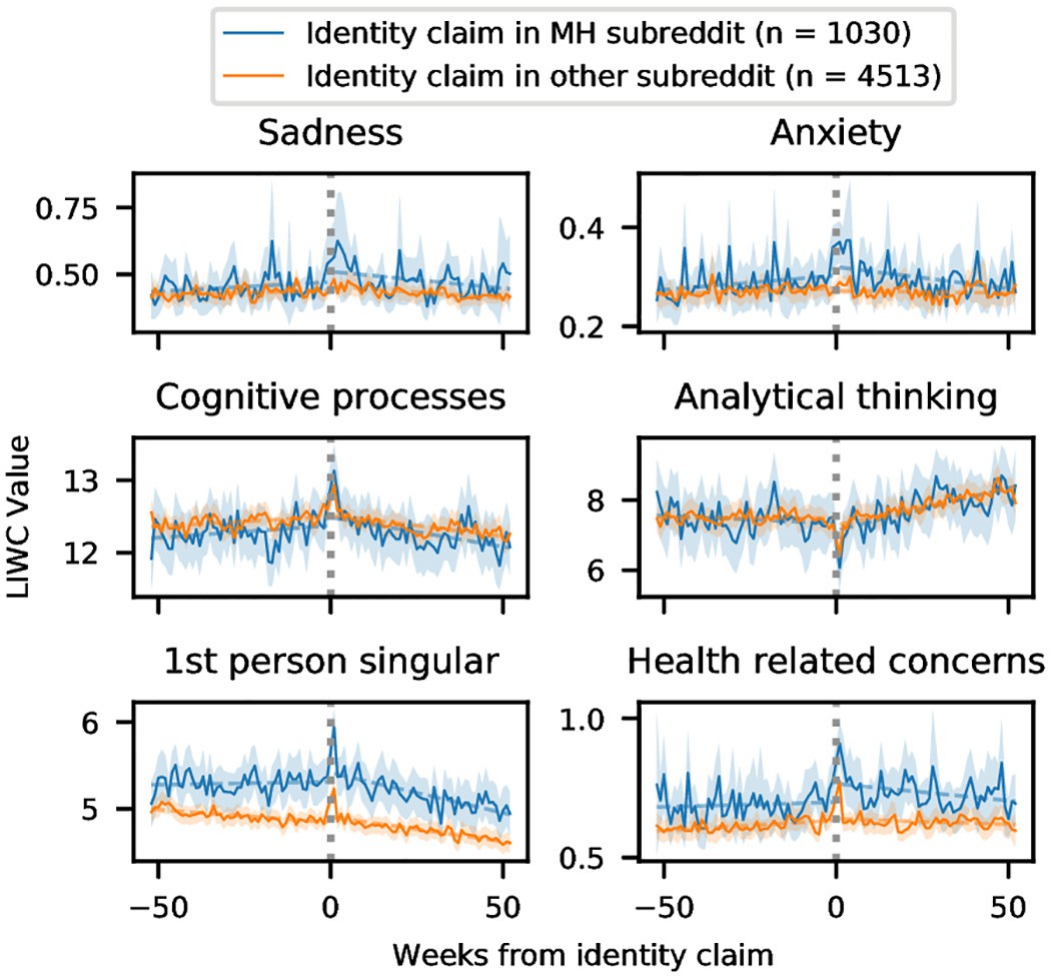
LIWC changes over time for users with whose identity claim takes place in a mental health subreddit, compared with those whose identity claim takes place in a subreddit with a different topic. Analytical thinking is computed based on other categories; the other y axes are percentage of total words. The solid line marks the weekly mean values; the dashed line shows the trend lines for the before and after periods, and the shaded area covers the 95% confidence interval.

Next, we graph LIWC features over time, with users split into those whose identity claim was shared in a mental health subreddit from those whose identity claim was shared in another subreddit (e.g., r/AskReddit). The time series plots are presented in Fig 3, and the interrupted time series analysis is presented in the S2 Appendix. We again found that the group we hypothesized to have stronger intentionality (in this case, those whose identity claim is shared in a mental health subreddit) experienced more notable changes over time. The changes in both groups are identical with respect to the change in direction of slope, but the magnitude of change is more notable for those whose identity claim appears in a mental health subreddit. Both groups exhibit significant changes in all of the examined language dimensions. In other words: identity claims coincide with behavior change whether or not they are posted in a mental health subreddit, but the change is more pronounced when they are shared with an audience that is knowledgeable about the identity being discussed.

An interesting observation is that some linguistic categories either spike (cognitive processes, analytical thinking) or level off (anxiety, 1st person singular) for those whose identity claim appears in a mental health subreddit around the 30 week mark; it is possible that this points to the cyclical and recurrent nature of depression [53]. Additional analysis on interaction with mental health subreddits is presented in the S3 Appendix.

### Robustness check

Our results suggest a change in features closely related to depression symptoms around the time of an identity claim; however, there are possible explanations of the phenomena that have little to do with the identity claim itself. For instance, if someone has recently been diagnosed with depression, they are likely *beginning treatment* for the first time, which could cause a change in the metrics we study. To examine this distinct possibility, we use an automated process to analyze user’s posts to determine whether they explicitly talk about treatment, and we use a manual process to analyze whether or not their IC posts indicated a recent change to their treatment regimens.

To determine whether users mention treatment (beyond diagnosis itself), we perform an automated annotation procedure on posts within the one-month periods before and after user’s identity claims. The precise choice of one month is arbitrary, but is chosen to capture potential for discussion of treatment close to the time of the identity claim. Our procedure uses a list of n-grams that indicate psychological treatment such as “therapist” and “meds” to identify mentions of treatment; the full list is available alongside our released data, and includes names of common anti-depressants. We label a post as “mentioning tretment” if any of the n-grams are included in the post.

To measure the accuracy of this procedure, we manually annotate one hundred identity claim posts (50 labeled as mentioning treatment; 50 labeled as not mentioning treatment), and we find that the method yields a 10% false-positive rate and a 24% false-negative rate. Following the manual annotation, we add 9 terms to the lexicon to reduce the false-negative rate.

With the final lexicon, we find that 83% of our IC users mentioned treatment within one month of their identity claim, compared to only 27% of control users. However, our primary goal is to determine whether or not a *change* in treatment linked to the user’s diagnosis is a likely cause of the changes that we observe in our analysis, rather than whether the user is receiving ongoing treatment or received treatment a number of years ago. To determine this, we manually re-annotate the 54 IC posts that we had annotated as mentioning treatment (the 46 posts that do not mention treatment by definition do not mention a change in treatment). The labels given to each post indicate whether or not the post mentions a *change in treatment* (defined as moving from one medication to another, starting treatment or plans to start treatment) or *ongoing/prior treatment* (defined as treatment that started well before the identity claim, whether or not it is still continuing).

We find that the majority of users who mentioned treatment at all (68.5%) spoke of ongoing or prior treatment. While the manual labeling is time-intensive and we are not able to label all of the posts, the results on the randomly selected sample suggest that the effects we observe are unlikely to be explained exclusively by an external change in user’s treatment regimens. However, we would still like to emphasize that due to the experimental setup, we are not able to determine a causational relationship between identity claims and alleviation of symptoms; our results are correlational, and we leave further analysis to future work.

## Discussion

Our results show that when identity claims occur, there are clear longitudinal shifts in language use; compared to control users, the users who make identity claims show substantially different patterns in language use, in which their behavior tends to deviate from the control users before the identity claim and become more similar afterwards. The singular instance in which the slope of the trend line did not change sign after the identity claim was for first person singular words; this is likely because they decrease over time in all online conversation, but we still observe a significant change in slope after the identity claim. As the language dimensions we focus on are typically indicative of depression, it is an encouraging result that the time after an identity claim leads to behavior that more clearly resembles controls, supporting the possibility of a therapeutic effect as was found in [13].

We find, however, that not all identity claims correspond with the same level of behavioral change; notably, identity claims that are made in Reddit *posts* are surrounded by more substantial changes in language use than those that occur in *comments*. We suspect that this has to do with the forethought required to make an identity claim in a post; a user must believe that they have something meaningful to say, and believe that their identity is central to what they plan to share. Meanwhile, if another user were to ask for advice about how to support their brother who has social anxiety, one may make an identity claim referencing their depression to show solidarity, even if their diagnosis had not previously been top-of-mind. The fact that somebody else can incite an identity claim in a comment makes it unsurprising that prior to the making an identity claim in a comment, user’s behavior more closely mirrors controls.

Meanwhile, identity claims that occurred in mental health subreddits initially appeared to correspond with more prominent changes than those occurring in other subreddits. We hypothesized that this would be the case, as posting in a mental health support community signals support-seeking intention. However, we note that these users post significantly more in mental health subreddits overall, and especially in the periods before and after their identity claim, and that the changes are clearly minimized when excluding activity that occurs in mental health subreddits (S3 Appendix). The exception is in sadness, in which a significant decrease is observed even when considering non mental-health posts.

### Limitations

In finding our identity claims, we are limited by the patterns we use [6]. While the patterns are high precision, they may not be high recall, in that they do not cover all possible misspellings or ways to state that one is diagnosed with depression. We choose to focus on the identity claims that specifically mention a diagnosis because statements such as “I am depressed” can occur in varied contexts that have nothing to do with clinical depression. We also do not find statements such as “Having depression sometimes makes it hard to get out of bed in the morning (I’m diagnosed),” despite a clear mention of a diagnosis, since the way in which it is stated does not match our patterns. This means that we cannot make any strong claims that this is the *first* time that any user explicitly mentions their diagnosis (it is also possible that the user has made many such identity claims outside of the Reddit platform), and that our results only show that the effects occur surrounding one identity claim. Given that our study is based entirely on archived social media posts, our findings are correlational; a limitation of our study is that we cannot make causal claims about depression identity claims.

A further limitation is the source of our Reddit data. Prior work has shown evidence of gaps in the IDs in the Pushshift dataset [54], beyond those that are expected due to some subreddits being private. These gaps appear more during certain dates, e.g., in the early years of Reddit and during the Reddit blackout of 2015. It was reported by [54] that by 2016, the error rate had evened out to approximately 1% of missing data per month. This may affect our study in several ways. It is possible that there are missing ICs posts in our collection, and more users would be included in our study had that not been the case. Relatedly, it is possible that users were filtered out for not having enough data due to missing posts. Missing posts could also lead to imperfect matching with controls. Finally, if missing posts differ substantially in any of the LIWC categories we studied from those that are present, it is possible that some of our results would change. However, given the fact that the missing posts seem to be minimal and largely random (besides being more common in certain time periods), we expect that this does not have a large impact on our results.

Similarly, we know that posts are sometimes removed from Reddit, either from being deleted by the user themself or being removed by a moderator. We are unable to study the content of these posts and ignore them entirely in our analysis. We acknowledge that it’s possible that these posts differ from the user’s other posts in some notable way (e.g., they might be more emotional or personal), and their exclusion, while it is ethically sound and technically necessary, may have some impact on our findings.

Finally, compared to the U.S. population, Reddit is disproportionately male, young, and liberal [55]. This means that the behavior of Reddit users might not be reflective of the general population. We seek out users with posts over a long period of time, which means that we likely capture behavior of the 1% of “superusers” who generate a large percentage of content on social media, and do not capture the 9% of social media users who post less frequently, or users who only read but do not generate any content [56].

### Ethical considerations

While it is valuable to expand our understanding of mental illness through large-scale data analysis, there are ethical concerns accompanying this line of research that are important to consider. Although the data that we use is publicly available, the users in our dataset posted on Reddit without the expectation that their content would be used for research purposes. Therefore, we follow a number of protocols proposed by [27], including paraphrasing examples included in this paper, replacing usernames with unique IDs during our data collection, and sharing features obtained after pre-processing, so that one cannot search for text to identify the usernames of users in our dataset. While we believe that it is not impossible to identify users with this data, for example by comparing LIWC features from a user’s posts to all users on Reddit, we believe that doing so would be significantly more complex than following our initial data collection procedure, which minimizes our concerns.

There are a number of additional ethical concerns that arise when researchers use social media data to build a model that *predicts* mental health states, including use by bad actors, performance, and interpretability [57]. While this is not a task that we explore in our work, there is potential for our dataset to be used for this task, because we include users who have diagnosed depression alongside matched controls. This magnifies the importance of anonymizing users.

### Future work

The causes of the language changes that we observe in our analysis are largely unknown, and leave interesting questions for future work. It is likely that the changes are connected to a number of factors that differ between users, including but not limited to direct benefits of posts including identity claims as a form of expressive writing [58], useful advice given in comments that improves the user’s mental state, and the formation of a supportive community that might persist beyond the original Reddit thread. The effects may also be related to changes that happen offline around the time of the identity claim. We discussed the potential for some effects to be related to treatment, but it is also possible that identity claims online correspond with the user opening up in their life offline. This could be directly related to the online identity claim (e.g., commenters encourage the user who shared to open up to their spouse or friends), but it is also possible that the two events occur but are unrelated. There are numerous ways in which offline support could explain the changes that we observe.

Without direct contact with users, the most feasible areas for future work involve analysis of additional individual-level attributes of ICs, and how they correlate with the changes observed for users following their IC. Some metrics that could be considered in this analysis are upvotes on the post or comment containing the IC (as a proxy for level of support), sentiment of replies to the IC, timeframe of the diagnosis (e.g., whether the user mentioned that the diagnosis occurred one year or one day ago), and the extent to which the user continues to be involved with the community in which their IC was posted following the IC.

We limited our analysis of subreddit-level differences to mental health and other subreddits, but in future work, we could also consider how user behavior changes when ICs are posted in subreddits that are meant to give personal support, but not with a mental health focus (e.g., r/offmychest, r/overcoming, etc.).

## Conclusion

In this study, we collect a dataset of 5854 Reddit users who disclose their depression diagnosis on Reddit (which we refer to as an identity claim) along with matched control users. We use interrupted time series analysis to measure differences in language usage after their identity claim. We find that there are significant longitudinal changes in language use around the time of depression diagnosis identity claims; these changes are not present in the control population. Language use across six LIWC categories becomes more similar to controls following the identity claim. Furthermore, the effects are stronger in certain contexts, including identity claims made in a post and identity claims made in mental health subreddits. Furthermore, we find that the language use in identity claims made in different context is substantially different, as identity claims made in posts and in mental health subreddits indicate more help-seeking behavior. In future work, it would be interesting to study the effects of other’s reactions to identity claims, as it has been shown to be an important indicator for how beneficial they are [9]. It would also be intriguing to more closely examine at an individual level what factors of identity claims correlate with positive outcomes, and to consider how this topic can be studied in a causal manner.

## Supporting information

S1 Appendix Data and LIWC summary. Dataset summary and examples of words from the LIWC categories analyzed in the text.
(PDF)

S2 Appendix Interrupted time series results. Tables showing coefficients and p-values for interrupted time series analysis that is referenced in the paper.
(PDF)

S3 Appendix Mental health subreddit analysis. Additional analysis of results when including and excluding posts from mental health subreddits.
(PDF)
