# Supplementary material for: Emotional and cognitive changes surrounding online depression identity claims
Source: PLoS One. 2022 Dec 1;17(12):e0278179. doi: 10.1371/journal.pone.0278179 (PMC9714698; doi:10.1371/journal.pone.0278179)
Supplement: S1 Appendix — Dataset summary and examples of words from the LIWC categories analyzed in the text. (PDF) [file pone.0278179.s001.pdf]

Data and LIWC Summary

This appendix includes additional data statistics and example words from the LIWC categories studied in the main text.

Table 1: Metrics for IC and control users (full data and filtered). Besides the number of identity claims, all metrics are computed excluding posts with diagnosis identity claims. Where applicable, metrics are first averaged at a per-user level, then averaged across users. Numbers in parentheses represent the number of users for which the metric was zero or undefined, which are not included in averages. The filtered data represents only a 2-year period, while the unfiltered data represents user's full history on Reddit.

	Users	Posts	Comments	Chars (P)	Chars (C)	# IC	Activity (days)
All IC	52292	99.48 (6550)	1708.62 (2548)	666.95 (6550)	236.44 (2548)	1.22 (0)	1072.55 (2131)
Filtered IC	5854	94.11 (66)	2468.66 (0)	537.93 (66)	216.93 (0)	1.22 (0)	720.10 (0)
Control	5854	50.64 (268)	1412.57 (5)	416.73 (268)	185.23 (5)	N/A	718.58 (0)

Table 2: The LIWC categories used in our analysis; examples are taken from [1, 2]. Analytical thinking is computed by combining eight categories, two additive (++) (article, preposition) and six subtractive (−−) (personal pronoun, impersonal pronoun, auxilary verb, conjunction, adverb, negation).

LIWC Category	Examples
Cognitive processes	cause, know, ought
Analytical thinking	++: a, an, the, below
	−−: you, this, did, have, just, and, no
Sadness	crying, grief, sad
Anxiety	worried, fearful
Health related concerns	clinic, flu, pill
1st person singular	I, me, mine

References

1. Pennebaker JW, Boyd RL, Jordan K, Blackburn K. The development and psychometric properties of LIWC2015. The University of Texas at Austin; 2015. Available from: https://repositories.lib.utexas.edu/bitstream/handle/2152/31333/LIWC2015_LanguageManual.pdf.

2. Pennebaker JW, Chung CK, Frazee J, Lavergne GM, Beaver DI. When Small Words Foretell Academic Success: The Case of College Admissions Essays. PLOS ONE. 2015;9(12):1–10. doi:10.1371/journal.pone.0115844.
