# Supplementary material for: Emotional and cognitive changes surrounding online depression identity claims
Source: PLoS One. 2022 Dec 1;17(12):e0278179. doi: 10.1371/journal.pone.0278179 (PMC9714698; doi:10.1371/journal.pone.0278179)
Supplement: S2 Appendix — Tables showing coefficients and p-values for interrupted time series analysis that is referenced in the paper. (PDF) [file pone.0278179.s002.pdf]

Interrupted Time Series Results

Table 1: Results of ITS analysis for diagnosis identity claim users and control users. Statistical significance after FDR correction is shown as follows: * ($p < 0.05$), ** ($p < 0.01$), *** ($p < 0.001$). For users who make an identity claim, there are significant changes at the $p < 0.001$ level for all language categories; for the control users, there is only a significant change for sadness.

	Diagnosis identity claim			Control		
	coef	pvalue		coef	pvalue	
Cognitive processes	-9.35e-05	1.48e-11	***	-1.27e-05	2.13e-01	
Analytical thinking	2.31e-04	4.31e-17	***	3.37e-05	2.13e-01	
Sadness	-1.36e-05	1.38e-08	***	7.63e-06	1.45e-03	**
Anxiety	-6.73e-06	2.31e-05	***	-1.17e-06	5.95e-01	
Health	-1.39e-05	7.24e-06	***	-1.45e-06	6.51e-01	
1st person singular	-4.43e-05	4.74e-08	***	-1.68e-06	7.85e-01	

Table 2: Results of ITS analysis for identity claim post users and identity claim comment users. Statistical significance after FDR correction is shown as follows: * ($p < 0.05$), ** ($p < 0.01$), *** ($p < 0.001$). The results mirror each other in that there are significant changes in the same direction (based on the coefficients) for users with both identity claim posts and comments. However, the absolute value of the coefficients is higher for those with identity claim posts, mirroring the larger changes in slopes that we observed in the graphs.

	Identity claim post			Identity claim comment		
	coef	pvalue		coef	pvalue	
Cognitive processes	-1.17e-04	2.04e-05	***	-7.95e-05	2.90e-08	***
Analytical thinking	2.51e-04	1.66e-05	***	2.03e-04	3.47e-13	***
Sadness	-2.23e-05	5.33e-04	***	-1.23e-05	4.61e-07	***
Anxiety	-1.45e-05	1.06e-03	**	-4.23e-06	6.53e-03	**
Health	-1.42e-05	3.38e-02	*	-1.30e-05	5.49e-05	***
1st person singular	-8.52e-05	1.85e-05	***	-2.64e-05	5.48e-04	***

Table 3: Results of ITS analysis for identity claim in mh subreddit users and identity claim in other subreddit users. Statistical significance after FDR correction is shown as follows: * ($p < 0.05$), ** ($p < 0.01$), *** ($p < 0.001$). The results mirror each other in that there are significant changes in the same direction (based on the coefficients) for users with identity claims in mental health and other subreddits. However, the absolute value of the coefficients is higher for those with identity claims in mental health subreddits, mirroring the larger changes in slopes that we observed in the graphs.

	Identity claim in MH subreddit			Identity claim in other subreddit		
	coef	pvalue		coef	pvalue	
Cognitive processes	-1.15e-04	1.43e-05	***	-8.48e-05	1.35e-09	***
Analytical thinking	2.85e-04	2.16e-07	***	2.15e-04	9.55e-15	***
Sadness	-1.96e-05	3.15e-03	**	-1.24e-05	1.63e-07	***
Anxiety	-1.46e-05	2.62e-04	***	-3.22e-06	3.37e-02	*
Health	-1.55e-05	2.37e-02	*	-1.12e-05	3.12e-04	***
1st person singular	-9.87e-05	1.43e-08	***	-2.73e-05	3.12e-04	***
