# Supplementary material for: Emotional and cognitive changes surrounding online depression identity claims
Source: PLoS One. 2022 Dec 1;17(12):e0278179. doi: 10.1371/journal.pone.0278179 (PMC9714698; doi:10.1371/journal.pone.0278179)
Supplement: S3 Appendix — Additional analysis of results when including and excluding posts from mental health subreddits. (PDF) [file pone.0278179.s003.pdf]

Mental Health Subreddit Analysis

Activity in subreddits that act as mental health support forums can be thought of as a proxy for a user's level of preoccupation with their mental illness. Therefore, we measure the percentage of posts that occur in such subreddits over time, using the list of subreddits from [1]. The results are shown in Figure 1. We find that those whose identity claim is in a mental health subreddit have vastly different activity levels in those subreddits over time than those whose identity claim takes place in another subreddit. For those with an identity claim in a mental health subreddit, activity level in mental health subreddits peaks directly following the identity claim, although it grows steadily up until the point of the identity claim. While it clearly decreases following the identity claim, it remains higher than it was during the first year, indicating that some newly active users may remain active in these communities to seek support during that year. For the other users, activity in these subreddits stays fairly constant at around 1% during the full two-year period.

In order to see if the changes we observe occur only in the mental health subreddit posts of those who make their identity claim in mental health subreddits, we plot results for the same LIWC categories for those users both including and excluding their mental health subreddit posts in Figure 2. We included users who did not match our longitudinal filtering threshold when only considering posts outside of mental health subreddits, as we saw no meaningful difference whether or not they were included. As is shown in the ITS analysis (Table 1), there are significant changes that occur when including mental health subreddit posts, but not when only considering other posts. The exception is sadness, for which the curves look almost identical, although the β_3 coefficient is still much smaller without mental health subreddit posts.

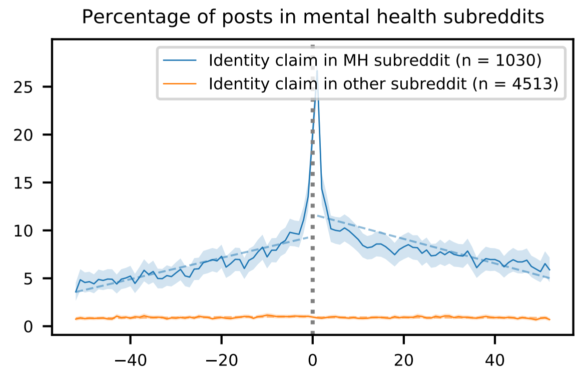

Figure 1: Percentage of posts that occur in mental health subreddits over time. We see that for those whose identity claims appear in mental health subreddits, the percentage of their posts that occur in those subreddits increases up to the time of the identity claim, where there is a sharp peak. After the identity claim, there is a decrease that almost mirrors the increase prior to the claim. For those whose identity claims appear in other subreddits, amount of activity in mental health subreddits remains constant over time.

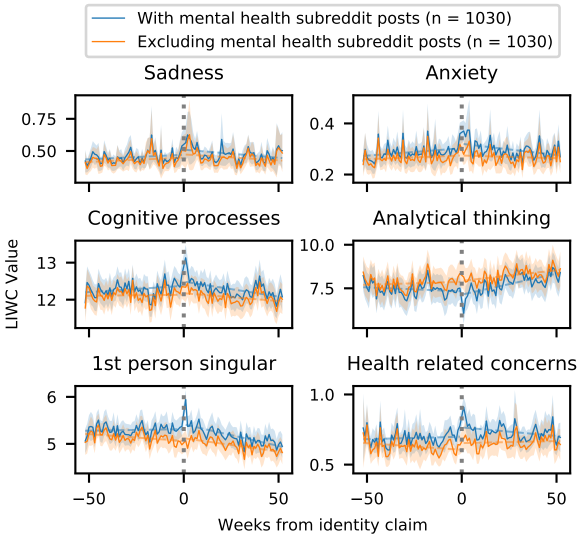

Figure 2: LIWC changes over time for users with whose identity claim takes place in a mental health subreddit; the blue line includes all of their posts, while the orange line excludes their posts that occur in mental health subreddits. Analytical thinking is computed based on other categories; the other y axes are percentage of total words. Across language categories, we see clearer changes in slope when including the mental health posts.

Table 1: Results of ITS analysis for with users with whose identity claim takes place in a mental health subreddit; "with mental health subreddit posts" includes all of their posts, while "excluding mental health subreddit posts" excludes their posts that occur in mental health subreddits (the same users are included in both cases). Statistical significance after FDR correction is shown as follows: * ($p < 0.05$), ** ($p < 0.01$), *** ($p < 0.001$). We see significant changes in all categories when including mental health subreddit posts, but only in sadness when those posts are excluded.

	With mental health subreddit posts			Excluding mental health subreddit posts		
	coef	pvalue		coef	pvalue	
Cognitive processes	-1.15e-04	2.85e-05	***	-4.75e-05	5.27e-02	
Analytical thinking	2.85e-04	5.40e-07	***	8.71e-05	7.26e-02	
Sadness	-1.96e-05	6.29e-03	**	-1.59e-05	2.46e-02	*
Anxiety	-1.46e-05	4.59e-04	***	-5.01e-06	1.53e-01	
Health	-1.55e-05	3.73e-02	*	1.22e-05	6.62e-02	
1st person singular	-9.87e-05	4.28e-08	***	-2.75e-05	5.27e-02	

References

1. Cohan A, Desmet B, Yates A, Soldaini L, MacAvaney S, Goharian N. SMHD: a Large-Scale Resource for Exploring Online Language Usage for Multiple Mental Health Conditions. In: Proceedings of the 27th International Conference on Computational Linguistics. Santa Fe, New Mexico, USA: Association for Computational Linguistics; 2018. p. 1485–1497. Available from: https://www.aclweb.org/anthology/C18-1126.
